# Effect of metformin exposure on growth and photosynthetic performance in the unicellular freshwater chlorophyte, *Chlorella vulgaris*

**DOI:** 10.1371/journal.pone.0207041

**Published:** 2018-11-12

**Authors:** Brittany M. Cummings, Joseph A. Needoba, Tawnya D. Peterson

**Affiliations:** Oregon Health & Science University–Portland State University School of Public Health, Oregon Health & Science University, Portland, Oregon, United States of America; Southern Cross University, AUSTRALIA

## Abstract

Many pharmaceuticals have negative effects on biota when released into the environment. For example, recent work has shown that the commonly prescribed antidiabetic drug, metformin (*N*,*N*-dimethylbiguanide), has endocrine disrupting effects on fish. However, effects of metformin on aquatic primary producers are poorly known. We exposed cultured isolates of a freshwater chlorophyte, *Chlorella vulgaris*, to a range of metformin concentrations (0–767.9 mg L^-1^) to test the hypothesis that exposure negatively affects photosynthesis and growth. A cessation of growth, increase in non-photochemical quenching (NPQ, NPQ_max_), and reduced electron transport rate (ETR) were observed 24 h after exposure to a metformin concentration of 767.8 mg L^-1^ (4.6 mM). By 48 h, photosynthetic efficiency of photosystem II (F_v_/F_m_), α, the initial slope of the ETR-irradiance curve, and E_k_ (minimum irradiance required to saturate photosynthesis) were reduced. At a lower concentration (76.8 mg L^-1^), negative effects on photosynthesis (increase in NPQ, decrease in ETR) were delayed, occurring between 72 and 96 h. No negative effects on photosynthesis were observed at an exposure concentration of 1.5 mg L^-1^. It is likely that metformin impairs photosynthesis either through downstream effects from inhibition of complex I of the electron transport chain or via activation of the enzyme, SnRK1 (sucrose non-fermenting-related kinase 1), which acts as a cellular energy regulator in plants and algae and is an ortholog of the mammalian target of metformin, AMPK (5' adenosine monophosphate-activated protein kinase).

## Introduction

Pharmaceuticals and personal care products (PPCPs) comprise a diverse class of chemical compounds that have gained attention as chemicals of emerging concern (CECs) due to their widespread detection in aquatic environments [1–3] and because of their bioactive properties [4–6]. Although the ecological effects of many active pharmaceutical ingredients (APIs) are poorly known, several have been implicated in acute [7–8] and chronic toxicity [9–11] to biota. Direct toxic effects of APIs on microalgae have been observed, with effects varying according to the species and drug [12–16].

The biguanide drug, metformin (*N*,*N*-dimethylbiguanide), is the most commonly prescribed oral medication used to treat type II diabetes [17,18]. Since the incidence of type II diabetes is on the rise worldwide [19], there are increasing reports of metformin detections in wastewater effluent and surface waters of North America [1,20, 21] and Europe [22–24]. The widespread detection of metformin is due in part to its unique biochemistry and mode of action. Although the mechanisms are not completely understood [25–27], metformin counteracts hyperglycemia through activation of AMP-activated protein kinase (AMPK) [28] by inhibition of complex I of the mitochondrial electron transport chain [29]. AMPK belongs to a class of energy sensing enzymes responsible for maintaining energy homeostasis; specifically, AMPK promotes energy releasing (catabolic) processes and downregulates energy depleting (anabolic) processes (e.g., synthesis of fatty acids, proteins) in response to an increase in adenosine monophosphate (AMP) relative to adenosine triphosphate (ATP) levels (i.e., adenylate charge), and increases catabolic processes (e.g., glycolysis) [30]. An increase in adenylate charge signals a decrease in gluconeogenesis and an increase in glycolysis [26, 28]. Metformin remains chemically unaltered during this process [31].

In the laboratory, fathead minnows exposed to metformin at levels observed in wastewater effluent (40 μg L^-1^) showed significant upregulation of mRNA encoding the production of vitellogenin [32], with stronger estrogenic effects seen in juvenile fish compared to adults [33]. This suggests that metformin may have negative effects on aquatic ecosystems, but effects on the lower aquatic food web are poorly known. Given its role as an AMPK-activator, however, metformin likely influences cell metabolism broadly among eukaryotes and would be therefore be expected to have widespread effects on the ecosystem. Limited data on the influence of metformin on phytoplankton suggest that lethal effects would not be expected at typical environmental levels concentrations < ~80 μg L^-1^ (with the highest values observed downstream of wastewater treatment plants [20, 24]) [12]; however, more subtle effects may occur, from direct effects such as suppression of photosynthesis or changes in species composition [13] due to chronic exposure, to indirect effects, such as the preferential elimination of grazers and trophic cascades [34].

The objective of this study was to determine the effects of metformin exposure on photosynthesis and growth in the freshwater chlorophyte, *Chlorella vulgaris*, isolated from the Columbia River, USA. This organism was chosen because it is a cosmopolitan freshwater taxa and its response to exposure should be broadly relevant. Moreover, as a chlorophyte, it shares similarities to land plants in terms of its photosynthetic machinery owing to a common evolutionary pathway [35]. Since photosynthesis is a highly regulated process in algae that enables cells to acclimate to fluctuating light environments, it was expected that responses in photosynthetic characteristics would be observed before changes in growth. The data will aid in characterizing toxicological effects of metformin exposure on AMPK/SnRK1/SNF1-containing eukaryotes in aquatic environments.

## Materials and methods

### Chemicals and materials

All glassware used in media preparation and culturing was cleaned according to the USGS National Water-Quality Assessment (NAWQA) protocols to minimize contamination from trace organic and inorganic constituents [36].

Metformin standards (as metformin hydrochloride) for the analysis of concentrations in growth media (0–1000 mg L^-1^) were prepared by dilutions of a primary metformin stock solution (10 mg mL^-1^ dissolved in mineral water; Toronto Research Chemicals). Mobile phase solvents included 0.1% UPLC-grade formic acid in HPLC-grade water (Sigma-Aldrich; solvent A), UPLC-grade 0.1% formic acid in HPLC-grade acetonitrile (Sigma-Aldrich; solvent B). Aliquots of 0.2 μm-filtered water were dispensed into 1.5 mL (12 x 32 mm) amber borosilicate glass SUN-SRI standard opening auto-sampler vials (Thermo Scientific). Internal standard (IS) solutions of deuterated metformin (Toronto Research Chemicals) were prepared from frozen stock solutions (1 mg mL^-1^ in Milli-Q water) diluted to yield a working solution (of 998 ng mL^-1^). Internal standard (10 μg L^-1^) was added to each sample and standard.

### Experimental design

Isolates of *C*. *vulgaris* from the Columbia River (1.8–5.1 μm in diameter) were grown in triplicate batch cultures in 250 mL glass Erlenmeyer flasks in WC medium [37] at 18 °C under a 12:12 light:dark cycle in a walk-in environmental chamber equipped with full-spectrum lighting (~191±18 μmol photons m^-2^ s^-1^). Cell densities were determined at each time point using a Beckman Coulter Model Z2 particle counter (Beckman Coulter, Indianapolis, IN) after dilution with Isoton II phosphate-buffered saline (Beckman Coulter, Indianapolis, IN). Cells were kept in suspension by continuous gentle shaking (80 rpm) to prevent negative effects of high biomass such as CO_2_ limitation of growth [38].

Triplicate cultures were grown to mid-exponential phase before receiving one of three target metformin concentrations: 1.5 mg L^-1^ (9.1 μM), 76.8 mg L^-1^ (0.46 mM), and 767.8 mg L^-1^ (4.6 mM), plus controls (0 mg L^-1^; no metformin added). These concentrations were selected to provide a broad range for the study of effects on photosynthesis. In addition, although the concentrations were much higher than would be observed in nature, the density of cells in culture was also orders of magnitude higher than in natural samples; thus, the average (or nominal) exposure by any given cell in the population could be considered to be approximately similar to environmental concentrations (e.g., the nominal concentration was the quotient of added concentration/cell density multiplied by the average cell density observed in a river sample [~4000 cells mL^-1^], yielding cell-specific exposure values of 11.5 ng L^-1^, 80 μg L^-1^, and 500 μg L^-1^). This was done to alleviate potential suppression of toxicity that can occur when high cell densities are used in bioassays [39]. A 1 mL pooled sample (i.e., 0.33 mL from each of three replicates) was taken at 0, 5, and 96 h to measure dissolved metformin concentrations in the medium to confirm the quantities added. The samples were filtered and frozen at -20°C prior to analysis by LC-MS/MS. An additional flask was included in each treatment and control to record pH over the course of the experiment. The initial pH was 7.3 ±0.017.

Cell density was determined in each treatment every 24 h (i.e., 0, 24 h, 48 h, 72 h, and 96 h). Photosynthetic performance was determined at shorter time intervals (0, 1, 3, and 5 h) following metformin addition, and then every 24 h for 96 h, since changes in photosynthesis are more likely to be observed over shorter time intervals than are changes in growth. Photosynthetic performance was characterized using a Water-PAM (Pulse-Amplitude-Modulation) chlorophyll fluorometer (Heinz Walz, Germany) according to parameters shown in Table 1. All samples taken for PAM fluorometry were diluted to 3 mL with WC media and dark-adapted for 30 min to fully oxidize Photosystem II and maximize fluorescence potential upon light saturation. Dark-adapted samples were exposed to 10 s of far-red light just before PAM measurements to process remaining inter-system electrons through excitation of Photosystem I and oxidation of the plastoquinone pool [40–42]. A 4-min light curve (where fluorescence of cells exposed to a saturating light pulse is measured at increasing levels of actinic light) was recorded for each dark-adapted sample.

**Table 1:** Photosynthetic parameters measured or calculated by PAM fluorometer.

Parameter	Notation		Determination method	Description
Maximum fluorescence	_Fm_ $F_{m}^{\prime }$		Direct measurement	Fluorescence when PSII reaction centers are closed and plastoquinone pool is reduced
Minimum fluorescence	F_o_		Direct measurement	Fluorescence when PSII reaction centers are open and plastoquinone pool is oxidized
Maximum quantum yield of PSII	ΦPSII_max_		$\frac{F_{v}}{F_{m}}\;or\;\frac{F_{m}-F_{o}}{F_{m}}$	Efficiency of dark-adapted PSII to absorb light
Effective quantum yield of PSII	ΦPSII		$\frac{F_{v}^{\prime }}{F_{m}^{\prime }}\;or\;\frac{F_{m}^{\prime }-F}{F_{m}^{\prime }}$	Efficiency of PSII in the presence of light
Relative electron transport rate	ETR		ΦPSII x PAR^a^ x 0.5 x 0.84	Rate of electron movement in the photosynthetic ETC^b^
Electron transport efficiency	α		Initial slope of light curve	Efficiency of electron transport in the photosynthetic ETC
Minimum saturating irradiance	E_k_		$\frac{{ETR}_{max}}{\alpha }$	Irradiance at onset of light saturation
Non-photochemical quenching	NPQ	$\frac{F_{m}-F_{m}^{\prime }}{F_{m}^{\prime }}$		Excitation dissipated by heat

^a^PAR = photosynthetically active radiation

^b^ETC = electron transport chain

Light saturation was determined by the maximum fluorescence (F_m_) resulting from the initial saturating pulse and subsequent fluorescence peaks (F_m_’) associated with the stepwise actinic pulses. Maximum quantum yield of Photosystem II (ΦPSII_max_, or F_v_/F_m_), relative electron transport rate (ETR), light intensity at saturation (E_k_), and non-photochemical quenching (NPQ) were calculated and considered indicators of photosynthetic performance [43, 44].

### Determination of metformin concentrations

Following a 10 μL injection, metformin was separated using a Shimadzu Prominence HPLC system using two binary pumps (Shimadzu LC-20AD XR Prominence LC pumps) and gradient elution on the reverse-phase column (Synerg Hydro-RP LC Column (250 x 4.6 mm, 4 μm, 80 Å; Phenomonex, Torrance, CA). A ThermoFisher (Waltham, MA) BetaBasic C8 Javelin guard column (10 x 2.1 mm, 1.5 μm) minimized column contamination from particulate matter in samples. The analyte was identified, detected, and confirmed using an AB Sciex QTRAP 5500 mass spectrometer (Applied Biosystems/MDS Sciex Instruments, Concord, ON, Canada) and Analyst 1.6.2 software. Samples with metformin concentrations >75 mg L^-1^ were diluted 1:500 in mineral water to improve peak resolution. The concentration of metformin was calculated from a standard curve of measured area ratios (analyte area/IS area) versus expected concentration ratios (analyte concentration/IS concentration). A single six-point linear standard curve with a 1/x weighting factor was established for each run.

### Statistics

Curves generated from ETR data were fit by non-linear regression using Microsoft Excel Solver; curve fitting for growth data was performed using GraphPad Prism (version 7.04, GraphPad Software, La Jolla, California). The resulting models were used to estimate parameters of growth and photosynthesis and differences among treatments and controls over time were tested using two-way repeated-measures ANOVAs performed using Microsoft Excel 2016 or GraphPad Prism.

Cell count data for short-term responses (0–5 h) to metformin addition were plotted against time and fitted with an exponential growth equation:

$${\text{N}}_{\text{t}}={\text{M}}_{\text{o}}\;{\text{e}}^{\mu \text{t}}$$ (1)

where N_t_ is the number of cells at time, *t*, N_0_ is the initial number of cells, and μ is the specific growth rate. Cell count data for the 0–96 h time period were plotted against time and fitted with a Weibull growth curve model (5) [45]. In addition to N_t_, N_0_, and μ, the time corresponding to the point of inflection (δ) and N_max_ (the maximum number of cells) are included in the Weibull model:

$$N_{t}=N_{max}-(N_{max}-N_{0})e^{\left(-{\left(\mu t\right)}^{\delta }\right)}$$ (2)

Specific growth rates at each exposure level and time point were evaluated for differences relative to controls according to a two-way ANOVA.

ETR data were fitted according to Platt et al. [46] and photosynthetic parameters including the initial slope of the ETR curve (α), slope of the photoinhibition region (β), minimum saturating irradiance (E_k_), and ETR_max_ were determined from the fitted data. Resulting models were used to compare parameter values among exposure levels and time points.

$$\text{ETR}={\text{ETR}}_{\text{max}}\left[1-\text{exp}\left(-\alpha \text{I}/{\text{ETR}}_{\text{max}}\right)\right]\left[\text{exp}\left(-ß\text{I}/{\text{ETR}}_{\text{max}}\right)\right]$$ (3)

Maximum quantum yield of PSII (ΦPSII_max_) and NPQ were compared among exposure levels and time using two-way repeated-measures ANOVAs.

## Results

### Effects of metformin on cell density and growth

**Fig 1:**
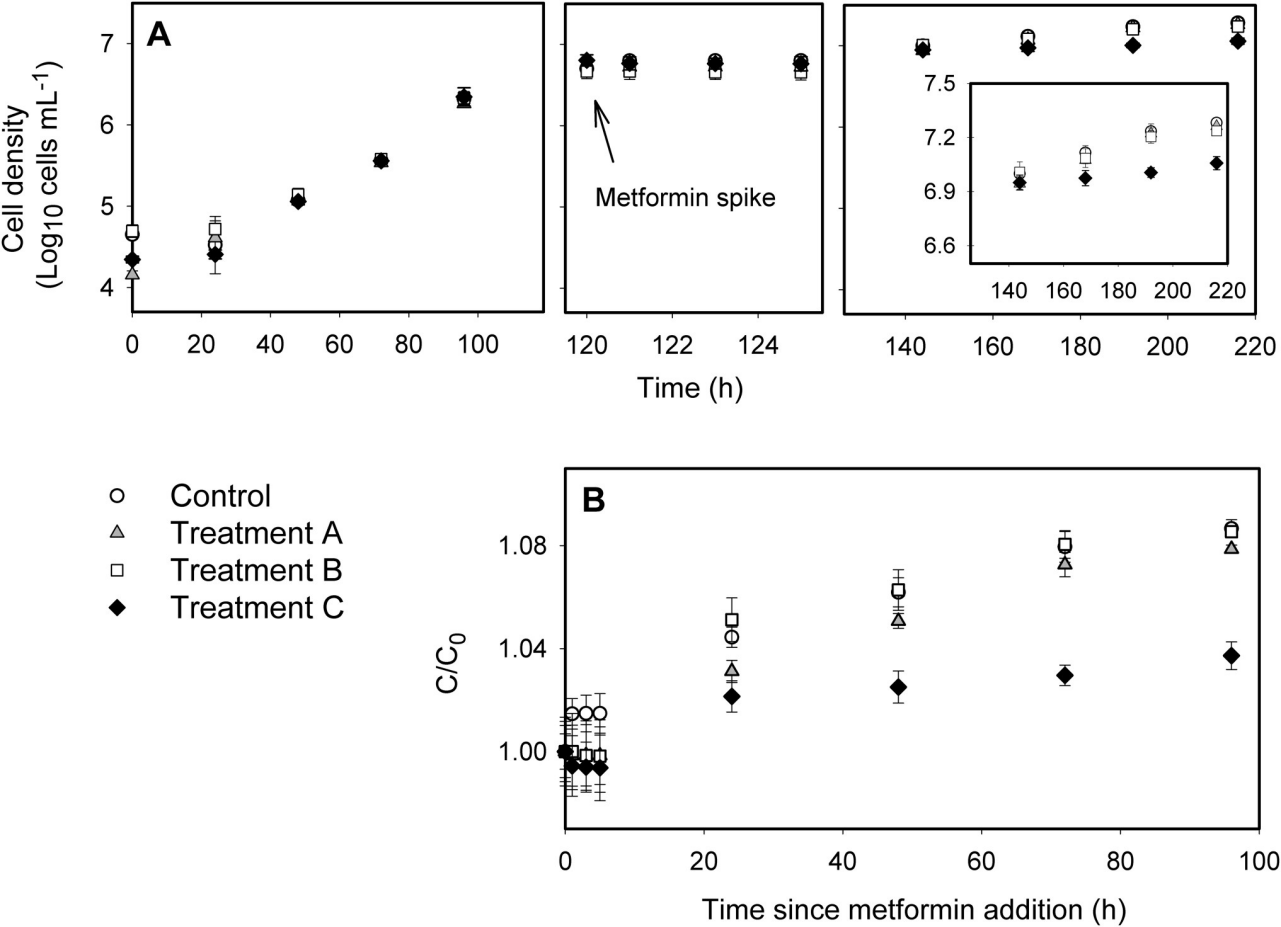
Algal growth curves (in log values) at different metformin concentrations. (A) Culture densities in *Chlorella vulgaris* in four triplicate cultures (three treatments and a control); first panel tracks cell densities over 96 h prior to a spike of three concentrations of metformin at 120 h, indicated in the second panel. Short-term changes in cell density were tracked for 5 h (120–129 h). Third panel shows cell densities at 144 h (24 h post-spike), 168 h (48 h post spike), 192 h (72 h post-spike), and 216 h (96 h post spike). (B) Ratio of cell density determined after inoculation relative to initial value (C/C_0_, where C_0_ = initial concentration at time = 120 h). Error bars represent ± 1 standard deviation.

Metformin concentrations remained within 20% of initial spike concentrations during the experiment (data not shown). Metformin reduced the culture density of *C*. *vulgaris* at exposures of 76.8 mg L^-1^ (0.46 mM) and 767.8 mg L^-1^ (4.6 mM) due to a rapid reduction in growth that was not observed in controls (Fig 1). There was a significant interaction between treatment and time (ANOVA, F(27,72) = 17.3, p<0.0001), whereby cell densities were reduced relative to controls by 48 h after an exposure of 767.8 mg L^-1^ (Tukey HSD). Unlike cell density, specific growth rates during the mid-exponential growth phase did not differ from the controls at any exposure concentration (data not shown), suggesting that growth ceased in response to exposure, rather than slowed.

The culture pH varied from 7.32 ±0.017 at T_0_ to 7.36 ±0.054 at T_72_ to 7.7 ±0.03 at the end of the experiment (T_166_); since pH did not increase beyond 7.7, it is unlikely that the cultures were limited by the availability of carbon (as carbon dioxide or bicarbonate) in the medium [47].

### Influence of metformin on photosynthetic performance

**Fig 2:**
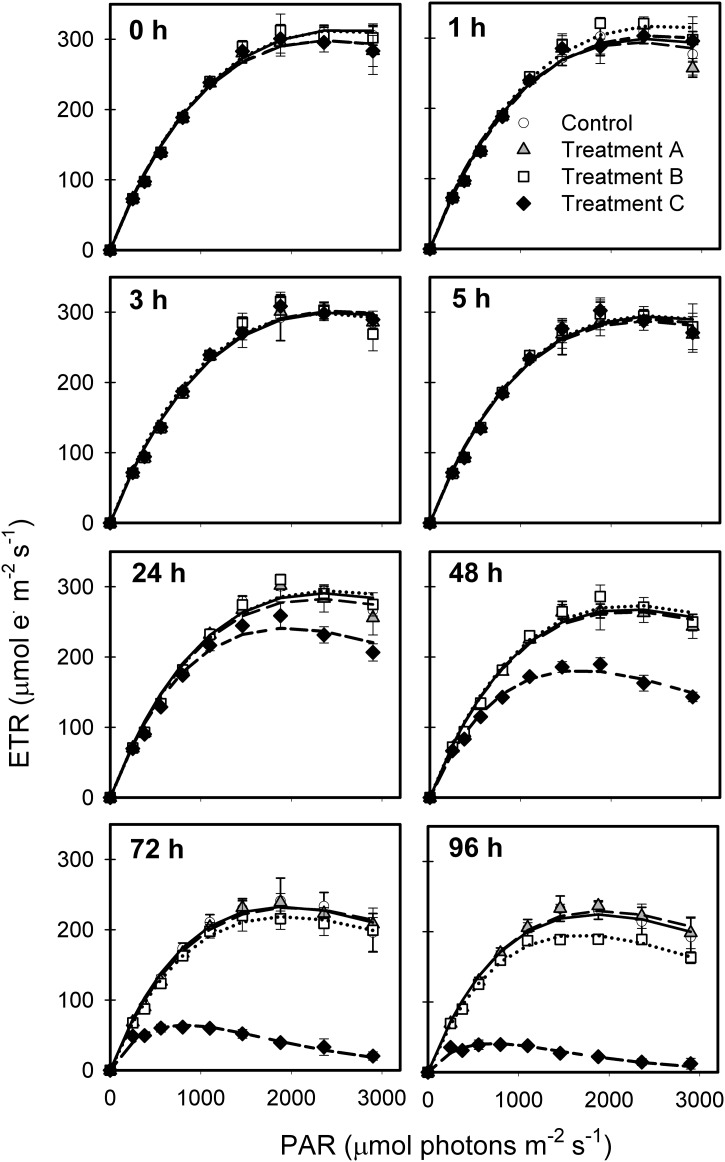
Effects of metformin on electron transport rates (ETR). Parameters were estimated by models of best fit according to Platt (1980) and include electron transport efficiency (α), photoinhibition (β), maximum relative electron transport rate (ETR_max_), and minimum saturating irradiance (E_k_). Symbols indicate different metformin additions: 0 μg L^-1^ (black circles), 1.5 mg L^-1^ (open squares), 76.8 mg L^-1^ (open diamonds), and 767.8 mg L^-1^ (open triangles).

**Fig 3:**
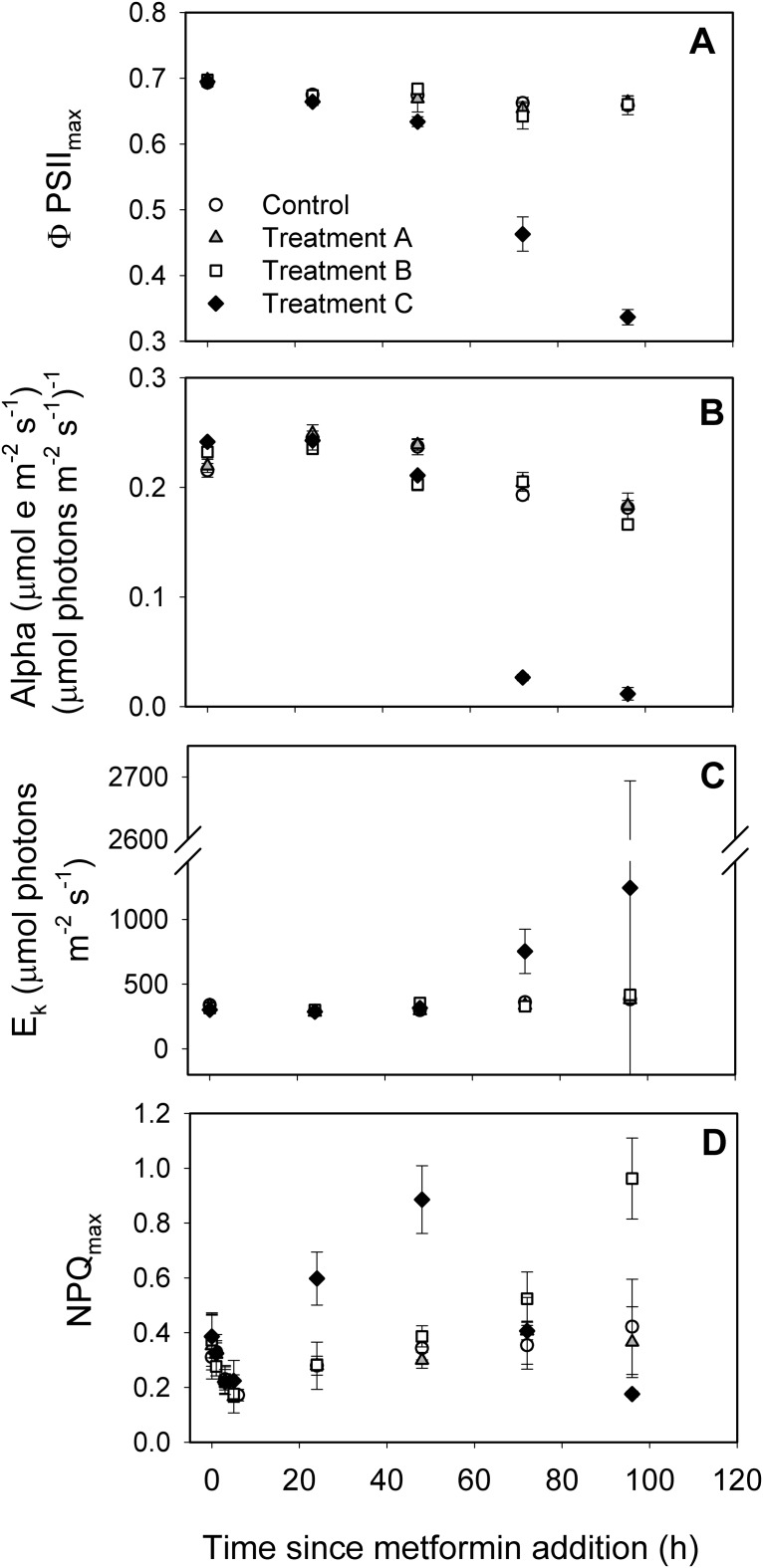
Effects of metformin on photosynthetic parameters of *C*. *vulgaris* over 96 h of exposure for four treatment levels: No addition (Control, open circles); 1.5 mg L^-1^ (Treatment A, open triangles); 76.8 mg L^-1^ (Treatment B, open squares); and 767.8 mg L^-1^ (Treatment C, closed diamonds). Parameters include (A) maximum yield of PSII (ΦPSII_max_), (B) electron transport efficiency (initial slope of the ETR curve, α), (C) minimum saturating irradiance (E_k_), and (D) maximum nonphotochemical quenching (NPQ_max_).

No short-term (<5 h) effects on electron transport rate (ETR)(Fig 2) or photosynthetic parameters (α, ΦPSII_max_, E_k_)(Fig 3) were observed at different exposure levels [ETR_max_, (F(12,32) = 0.8302, p = 0.6199); α (F(12,32) = 1.84, p = 0.082), ΦPSII_max_ (F(12,32) = 1.09, p = 0.401, and E_k_ (F(12,32) = 1.405, p = 0.2145)]. Significant variability in β across treatment and time (F(12,32) = 3.224, p = 0.004) appeared to be stochastic, with no systematic differences among any of the treatments when single effects were examined.

By 24 h, ETR_max_ was reduced in the 767.8 mg L^-1^ exposure (F(12,32) = 38.91, p<0.0001; Tukey HSD, p = 0.0065, p<0.0001) (Fig 2). Further decreases were observed until ETR reached baseline levels at 96 h, with the steepest decline occurring 48–72 h after exposure. At lower exposure levels, reduction of ETR_max_ was delayed or absent. At the 76.8 mg L^-1^ exposure level, ETR declined between 72 and 96 h (Tukey HSD, p = 0.007), while no negative effects on ETR were observed at the 1.5 mg L^-1^ exposure.

There were significant interactions between exposure concentration and α (F(12,32) = 40.02, p<0.0001) and exposure concentration and E_k_ (F(12,32) = 12.62, p<0.0001). Upon exposure to 767.8 mg L^-1^ metformin, α values were 10-fold lower than controls and other treatments (Tukey HSD, p = 0.0029) (Fig 3). By 24 h, average E_k_ values in the 767.8 mg L^-1^ exposure level were significantly lower than in the controls, decreasing from 885 to 263 μmol photons m^-2^ s^-1^ over 96 h (Tukey HSD, p = 0.0003). ΦPSII_max_ decreased >two-fold (~0.7 to 0.3 μmol photons^-1^ m^2^ s^-1^) by 48 h (p = 0.0005). A significant increase in NPQ_max_ was observed between 0 and 48 h at the highest exposure concentration (F(12,32) = 15.6, p<0.0001; Tukey HSD, p<0.0001). NPQ_max_ of the 76.8 mg L^-1^ exposure also increased with time, reaching ~0.9 by 96 h (Tukey HSD, p<0.0001).

**Fig 4:**
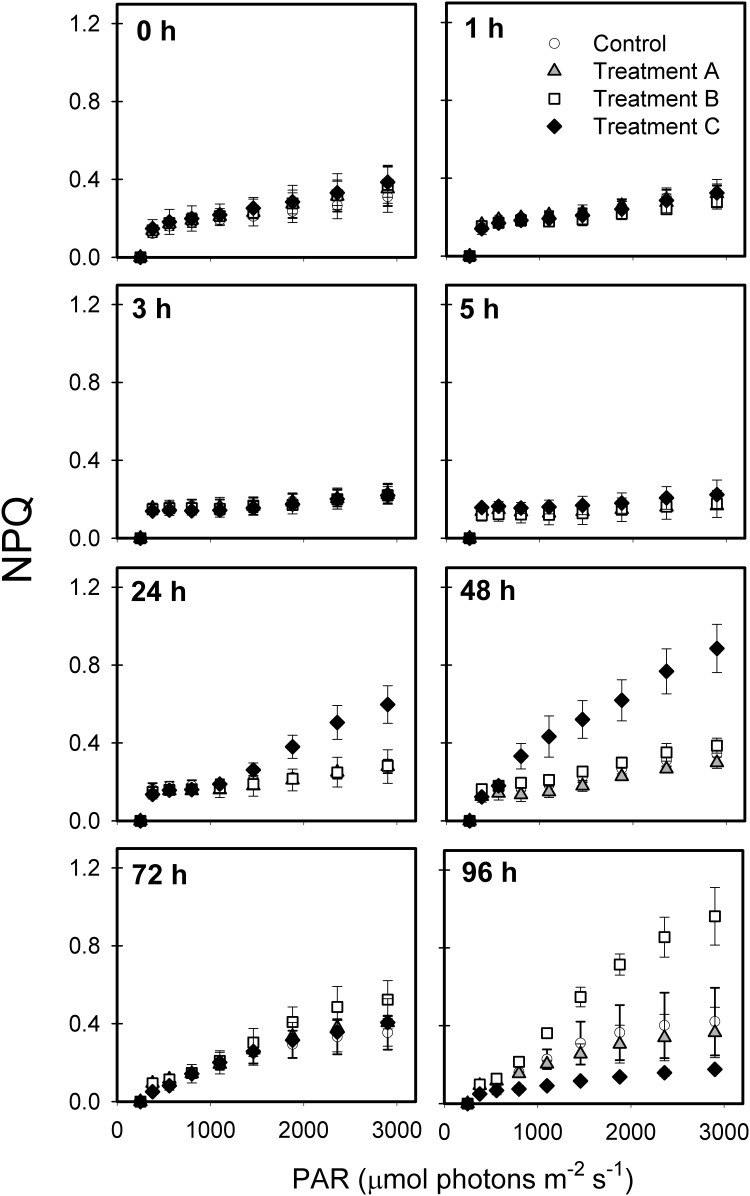
Non-photochemical quenching (NPQ) versus photosynthetically active radiation (PAR) after exposure to three levels of metformin (Treatment A = 1.5 mg L^-1^, Treatment B = 76.8 mg L^-1^, and Treatment C = 767.8 mg L^-1^) and a control over 96 h.

NPQ was elevated at irradiances >~1500 μmoL photons m^-2^ s^-1^ 24 h and 48 h after exposure to the highest metformin concentration relative to controls and to the other treatments (Fig 4). Between 48 and 72 h post-spike, NPQ declined in the highest exposure treatment, but increased in the 76.8 mg L^-1^ treatment at the higher irradiances. By 96 h, NPQ was lower in the highest-exposure cultures than the controls, while values exceeded controls in the 76.8 mg L^-1^ treatment for most irradiance levels (Fig 4).

## Discussion

Growth in *Chlorella vulgaris* ceased 24–48 h after exposure to 767.8 mg L^-1^ (4.6 mM) metformin, while exposure to lower concentrations (i.e., 76.8 mg L^-1^) led to delayed effects (72–96 h) on photosynthesis and growth, including an increase in non-photochemical quenching (NPQ), the dissipation of light energy not used to carry out photochemistry. Delayed effects on growth could reflect a shift to the use of internal carbon stores, as occurs under conditions of low or fluctuating irradiance [48] or it could occur if upregulation of membrane transporters is required for metformin to enter mitochondria where it inhibits complex I and activates AMPK [31, 49]. Indeed, metformin is known to accumulate within mitochondria of mammalian cells [50, 51] through active transport across the membrane down a chemiosmotic gradient via organic cation transporters (e.g., OCT1–3; [52]). The number of available transporters may thus dictate effective metformin concentrations found within mitochondria, which require time for upregulation and thus lead to delayed effects.

Photosynthetic performance in *C*. *vulgaris* was impaired 24 h after exposure at the highest concentration (767.8 mg L^-1^), shown by a ~20% decline in both electron transport rate (ETR_max_) and in the minimum irradiance capable of saturating photosynthesis (E_k_). These effects indicate a reduced capacity to process light energy, which is also supported by the observed decline in α and F_v_/F_m_ (after 48 h) and most dramatically in elevated NPQ and NPQ_max_ values (after 24 h). The depression in ETR_max_ observed in *C*. *vulgaris* compared to controls is reminiscent of the response to low light in a number of algal species, where light-limited cells reach a much lower maximum rate of photosynthesis compared to those grown at saturating irradiance [53–55]. A decrease in F_v_/F_m_ is generally interpreted as a signal of physiological stress in unialgal cultures; F_v_/F_m_ is also typically depressed under high light [56]. To date, effects of metformin on photosynthesis have not been shown; the unusual biochemistry of the compound makes it somewhat difficult to predict its physiological target in aquatic microorganisms, since there are no chemical analogues (aside from other biguanide compounds) with known toxic modes of action with which to compare effects as has been done with other pharmaceutical compounds such as fluoxetine [57].

Non-photochemical quenching describes a number of processes involved in energy dissipation and photoprotection in photosynthetic cells [58]. NPQ components—differentiated by relaxation kinetics in response to a saturating pulse of light—include qE, a rapid-response de-excitation mechanism driven by the pH of the chloroplast lumen, qT, quenching that accompanies state transitions (a shift in the location of light harvesting antennae complexes from the readily fluorescent photosystem II to the weakly fluorescent photosystem I), and qI, or photoinhibitory quenching, which is associated with either protein damage or the reversible down-regulation of photosystem II, sometimes referred to as qRC (where RC refers to reaction center [59]). Among these, the likely origin of the response by *C*. *vulgaris* to metformin exposure in this study is qI. The rationale behind this hypothesis is that the timescale of response by qE is short (seconds); the timescale for qT is slower, but still on the order of minutes. On the other hand, quenching that occurs at the reaction center is slower (hours). The fact that changes in NPQ and NPQ_max_ occurred over a period of days in the present study suggests a longer-term loss of capacity to process light energy. In addition, it has been shown that qE deficient mutants of *Chlamydomonas reinhardtii* are able to acclimate to growth under constant—but not fluctuating—conditions [60]; in contrast, growth was impaired in the present study.

The increase in NPQ over time in *C*. *vulgaris* in response to metformin exposure suggests that the cells were less able to process the same amount of light energy over time. qI results from a long-term downregulation of photosystem II, and likely occurs through a combination of photoprotection and photoinhibition [61]. In the present case, the increase in NPQ likely accompanied photoinhibition (and damage to photosystem II) rather than photoprotective quenching, given that a reduction in F_v_/F_m_ was observed. That is, F_o_ should decrease in direct proportion to F_m_ in the case of photoprotective quenching, while increased F_o_ is accompanied by decreased F_m_ when quenching is achieved through photoinhibition, thus reducing (F_m_-F_o_)/F_m_, or F_v_/F_m_ [61].

Interestingly, activation of SnRK1 (SNF1-related protein kinase 1)—an ortholog of AMPK, the target of metformin—is also associated with a decrease in the rate of photosynthesis in plants [62, 63]. Although not completely understood, SnRK1 activation occurs under energy deficit conditions, for example in darkness; to conserve energy, energetically demanding processes such as protein synthesis and cell division are inhibited [64, 65]. In contrast, SnRK1 is inhibited by sugars [66]. Recently, genes for SnRK1 were discovered in eukaryotic algae [67], and regulatory proteins involved in SnRK1 signaling pathways have been identified in both green algae and diatoms [68, 69]. Since energy sensing kinases are highly conserved among humans (AMPK), plants (SnRK1), and phytoplankton (SnRK1) [70, 71] the observed effects on growth and photosynthesis in *C*. *vulgaris* could be due to activation of SnRK1. It is possible that SnRK1 is implicated in stress responses to emerging contaminants such as pharmaceutical by algae more broadly, and therefore identifying factors that activate SnRK1 pathways would be valuable in assessing ecosystem effects of PPCPs.

Alternatively, it is possible that metformin inhibits complex I of the electron transport chain within algal mitochondria, resulting in a suppression of photosynthesis due to a reduction in interactions between organelles, either driven by inhibition of complex I or through activation of SnRK1. SnRK1 has been implicated in energy signaling between organelles [72]; in addition, a significant role for metabolic interactions between the chloroplast and the mitochondrion was demonstrated in mutants lacking complexes I and IV activity in another green alga, *Chlamydomonas*, which showed a 40% reduction in the light-saturated capacity of O_2_ evolution [73]. This reduction in the rate of photosynthesis is similar to the ~20% reduction in ETR_max_ in this study, although more dramatic, consistent with the idea that complex I may be a target in eukaryotic phytoplankton. Another example comes from the diatom, *Phaeodactylum tricornutum*, where upregulation of alternative oxidase (AOX) activity in the mitochondrion occurred in response to iron-deplete conditions [74] suggesting a possible way to reroute photosynthetic electrons towards respiration rather than photosynthesis [75].

The fact that inhibition of complex I by metformin is observed across different phylogenetic groups including the yeast, *Pichia pastoris*, the bacterium, *Escherichia coli*, and the bovine heart mitochondria [49, 51] indicates that the biguanide binding site is found in the core subunit that is conserved across taxa, not just in mammalian cells [76]. Although the reported effect concentrations tend to be substantially higher than those found in the extracellular matrix or ambient environment (e.g., IC_50_ of 22.6 ±4.3 mM in *P*. *pastoris* [49]), the 1000-fold accumulation of metformin within the mitochondrial matrix [51, 77] makes even low levels physiologically relevant. Moreover, in the environment, persistent exposure to metformin (for example in slow-moving reservoirs or lakes or else due to constant input fluxes [78]) could potentially lead to bioaccumulation and negative effects as transporters are synthesized. The issue of bioaccumulation is complex, since the multitude of pharmaceutically active ingredients with highly diverse chemistries show different propensities for bioaccumulation or biomagnification in the food web [79]. A recent study in Taihu Lake in China suggested that trophic magnification of several pharmaceuticals including norloxacin, ciproflaxin, and tetracycline are low [80, 81]. Persistent exposure to the antibiotic, ciproflaxin, downstream of a wastewater treatment plant in Kansas, United States, however, was associated with shifts in algal assemblages, a loss of genus richness, decrease in biomass yield, and changes in algal biovolumes [13].

In addition to deleterious effects on biota, exposure to chemical contaminants can lead to physiological acclimation (at sub-lethal concentrations) as well as evolutionary adaptation [82], which enables certain genotypes to survive, owing to the occurrence of beneficial mutations; this phenomenon is referred to as evolutionary rescue [83]. In the face of environmental deterioration, species–including microalgae [84, 85]–can become resistant to xenobiotics, which likely underlies shifts in algal assemblages observed downstream of wastewater treatment plants [13] as sensitive species are lost and resistant species persist. Evolutionary rescue appears to be strongest when effects are slow, with repeated exposure, and dispersal is modest [83]. Although exposures to low levels of a variety of pharmaceuticals have unknown consequences for aquatic biota [86], evolutionary rescue could be invoked in response to circumstance where risks are high, for example if chemical mixtures, particular life stages, or different physiological stressors render certain genotypes sensitive to exposure in ways that cannot be overcome by short-term physiological acclimation.

The present study is limited by the fact that effects of long-term exposures were not assessed; however, exposure to pharmaceutical compounds and other chemicals of emerging concern can occur over short or long time periods, making studies of both types necessary to evaluate ecological effects of unregulated contaminants. In addition, this study focuses on a single pharmaceutical compound in isolation, exposed to a single target organism; this clearly does not adequately represent natural environmental conditions [81], and therefore the results should be interpreted as such. Nevertheless, our approach was to determine whether exposure to an unusual pharmaceutical compound would elicit effects on photosynthesis in suspended algal populations, and the results shed some light on potential mechanisms.

Finally, recent evidence has shown that different biguanides [including antimalarial drugs, anti-hyperglycemic drugs (metformin, phenformin, and buformin), and drugs for the treatment of cardiovascular disease and cancer; [51]) inhibit complex I to varying degrees. We tested the effects of one biguanide on photosynthetic performance; it is possible that other biguanides could have different effects on complex I or in the activation of SnRK1, which could influence photosynthetic processes differently, meriting further investigation into their toxicological effects. Future work should explore the mechanisms by which SnRK1 activation occurs, as well as responses by SnRK1 to environmental toxicants in order to better understand broader impacts on photosynthetic processes.

## Supporting information

S1 Table Cell concentration data used to calculate experiment growth rates. Cell concentrations determined at each experimental time point and used to calculate specific growth rate. Cells were enumerated using a Coulter counter. Triplicate samples were averaged, and cell densities determined by accounting for dilution of the samples prior to enumeration.
(XLSX)

S2 Table Photosynthetic performance data showing response to metformin additions. Primary data produced by a Pulse Amplitude Modulated fluorometer for experimental time points. The data were collected for 120 h before spiking the cultures with three concentrations of metformin, plus a control. Photosynthetic characteristics were tracked for 1, 3, and 5 h after the spike and each 24 h thereafter (until 96 h after the spike). F = fluorescence; F_m_’ = effective maximum fluorescence; F_v_/F_m_ = yield of PSII; ETR = electron transport rate; PAR = photosynthetically active radiation; NPQ = nonphotochemical quenching. Primary ETR and NPQ data from light curves are also included.
(CSV)

## References

[1] Kolpin DW, Furlong ET, Meyer MT, Thurman EM, Zaugg SD, Barber LB, et al Pharmaceuticals, hormones, and other organic wastewater contaminants in U.S. streams, 1999–2000: A national reconnaissance. Environ. Sci. Technol. 2002; 36: 1202–12112. 11944670 10.1021/es011055j

[2] Boyd GR, Reemtsma H, Grimm DA, Mitra S. Pharmaceuticals and personal care products (PPCPs) in surface and treated waters of Louisiana, USA and Ontario, Canada. Sci. Tot. Environ. 2003; 311, 135–149. 10.1016/S0048-9697(03)00138-4 12826389

[3] Kim SD, Cho J, Kim IS, Vanderford BJ, Snyder SA. Occurrence and removal of pharmaceuticals and endocrine disruptors in South Korean surface, drinking, and waste waters. Water Res. 2007; 41: 1013–1021. 10.1016/j.watres.2006.06.034 16934312

[4] Le-Minh N, Khan SJ, Drewes JE, Stuetz RM. Fate of antibiotics during municipal water recycling treatment processes. Water Res. 2010; 44, 4295–4323. 10.1016/j.watres.2010.06.020 20619433

[5] Oulton RL, Kohn T, Cwiertny DM. Pharmaceuticals and personal care products in effluent matrices: A survey of transformation and removal during wastewater treatment and implications for wastewater management. J. Environ. Monit. 2010; 12(11): 1956–1978. 10.1039/c0em00068j 20938541

[6] Verlicchi P, Al Aukidy M, Galletti A, Petrovic M, Barceló D. Hospital effluent: Investigation of the concentrations and distribution of pharmaceuticals and environmental risk assessment. Sci. Tot. Environ. 2012; 430: 109–118. 10.1016/j.scitotenv.2012.04.055 22634557

[7] Hernando MD, Petrovic M, Fernández-Alba AR, Barceló D. Analysis by liquid chromatography-electrospray ionization tandem mass spectrometry and acute toxicity evaluation for β-blockers and lipid-regulating agents in wastewater samples. J. Chromatogr. A 2004; 1046: 133–140. 15387181

[8] Kim J-W, Ishibashi H, Yamauchi R, Ichikawa N, Takao Y, Hirano M, et al Acute toxicity of pharmaceutical and personal care products on freshwater crustacean (*Thamnocephalus platyurus*) and fish (*Oryzias latipes*). J. Toxicol. Sci. 2009; 34: 227–232. 19336980 10.2131/jts.34.227

[9] Crane M, Watts C, Boucard T. Chronic aquatic environmental risks from exposure to human pharmaceuticals. Sci. Tot. Environ. 2006; 367: 23–41. 10.1016/j.scitotenv.2006.04.010 16762401

[10] Brausch JM, Connors KA, Brooks BW, Rand GM. Human pharmaceuticals in the aquatic environment: A review of recent toxicological studies and considerations for toxicity testing. Springer 2012;, Boston, MA, pp. 1–99. 10.1007/978-1-4614-3137-4_1 22488604

[11] Guiloski IC, Stein Piancini LD, Dagostim AC, de Morais Calado SL, Favaro LF, Boschen SL, et al Effects of environmentally relevant concentrations of the anti-inflammatory drug diclofenac in freshwater fish *Rhamdia quelen*. Ecotoxicol. Environ. Saf. 2017; 139: 291–300. 10.1016/j.ecoenv.2017.01.053 28167441

[12] Cleuvers M. Aquatic ecotoxicity of pharmaceuticals including the assessment of combination effects. Toxicol. Lett. 2003; 142: 185–194. 12691712 10.1016/s0378-4274(03)00068-7

[13] Wilson BA, Smith VH, Frank deNoyelles J, Larive CK. Effects of three pharmaceutical and personal care products on natural freshwater algal assemblages. Environ. Sci. Technol. 2003; 37(9): 1713–1719. 12775039 10.1021/es0259741

[14] Andreozzi R, Caprio V, Ciniglia C, de Champdoré M, Lo Giudice R, Marotta R, et al Antibiotics in the environment: Occurrence in Italian STPs, fate, and preliminary assessment on algal toxicity of amoxicillin. Environ. Sci. Tech. 2004; 38(24): 6832–6838. 10.1021/es049509a 15669346

[15] Eguchi K, Nagase H, Ozawa M, Endoh YS, Goto K, Hirata K, et al Evaluation of antimicrobial agents for veterinary use in the ecotoxicity test using microalgae. Chemosphere 2004; 57, 1733–1738. 10.1016/j.chemosphere.2004.07.017 15519420

[16] Johnson DJ, Sanderson H, Brain RA, Wilson CJ, Solomon KR. Toxicity and hazard of selective serotonin reuptake inhibitor antidepressants fluoxetine, fluvoxamine, and sertraline to algae. Ecotoxicol. Environ. Saf. 2007; 67: 128–139. 10.1016/j.ecoenv.2006.03.016 16753215

[17] Bennett W, Wilson L, Bolen S. Oral diabetes medications for adults with type 2 diabetes: an update. Agency Healthcare Res. Qual. 2011; 1–320. 21735563

[18] Bailey CJ. Metformin: historical overview. Diabetologia 2017; 60: 1566–1576. 10.1007/s00125-017-4318-z 28776081

[19] World Health Organization. Global Report on Diabetes 2016; pp. 1–88.

[20] Bradley PM, Journey CA, Button DT, Carlisle DM, Clark JM, Mahler BJ, et al Metformin and other pharmaceuticals widespread in wadeable streams of the southeastern United States. Env. Sci. Tech. Rep. 2016; 3: 243–249.

[21] Meador JP, Yeh A, Young G, Gallagher EP. Contaminants of emerging concern in a large temperate estuary. Environ. Pollut. 2016; 213: 254–267. 10.1016/j.envpol.2016.01.088 26907702 PMC5509463

[22] Blair BD, Crago JP, Hedman CJ, Klaper RD. Pharmaceuticals and personal care products found in the Great Lakes above concentrations of environmental concern. Chemosphere 2013; 93, 2116–2123. 10.1016/j.chemosphere.2013.07.057 23973285

[23] Oosterhuis M, Sacher F, ter Laak TL. Prediction of concentration levels of metformin and other high consumption pharmaceuticals in wastewater and regional surface water based on sales data. Sci. Total Environ. 2013; 442: 380–388. 10.1016/j.scitotenv.2012.10.046 23183121

[24] Trautwein C, Berset JD, Wolschke H, Kümmerer K. Occurrence of the antidiabetic drug metformin and its ultimate transformation product guanylurea in several compartments of the aquatic cycle. Environ. Int. 2014; 70: 203–212. 10.1016/j.envint.2014.05.008 24954924

[25] Hur KY, Lee MS. New mechanisms of metformin action: Focusing on mitochondria and the gut. J. Diabetes Investig. 2015; 6: 600–609. 10.1111/jdi.12328 26543531 PMC4627534

[26] Viollet B, Guigas B, Garcia NS, Leclerc J, Foretz M, Andreelli F. Cellular and molecular mechanisms of metformin: an overview. Clin. Sci. 2012; 122: 253–270. 10.1042/CS20110386 22117616 PMC3398862

[27] Wang YW, He SJ, Feng X, Cheng J, Luo YT, Tian L, et al Metformin: A review of its potential indications. Drug Des. Devel. Ther. 2017; 11: 2421–2429. 10.2147/DDDT.S141675 28860713 PMC5574599

[28] Zhou G, Myers R, Li Y, Chen Y, Shen X, Fenyk-Melody J, et al Role of AMP-activated protein kinase in mechanism of metformin action. J. Clin. Invest. 2001; 108(8): 1167–1174. 10.1172/JCI13505 11602624 PMC209533

[29] Owen MR, Doran E, Halestrap AP. Evidence that metformin exerts its anti-diabetic effects through inhibition of complex I of the mitochondrial respiratory chain. Biochem. 2000; 348: 607–614. PMC1221104 10839993

[30] Hardie DG. AMP-activated/SNF1 protein kinases: Conserved guardians of cellular energy. Nat. Rev. Mol. Cell Biol. 2007; 8: 774–785. 10.1038/nrm2249 17712357

[31] Physicians Desk Reference, 71^st^ edition. PDR Network 2017; 2000 pp.

[32] Niemuth NJ, Jordan R, Crago J, Blanksma C, Johnson R, Klaper RD. Metformin exposure at environmentally relevant concentrations causes potential endocrine disruption in adult male fish. Environ. Toxicol. Chem. 2015; 34: 291–296. 10.1002/etc.2793 25358780 PMC4329414

[33] Crago J, Bui C, Grewal S, Schlenk D. Age-dependent effects in fathead minnows from the anti-diabetic drug metformin. Gen. Comp. Endocrinol. 2016; 232: 185–190. 10.1016/j.ygcen.2015.12.030 26752244

[34] Fleeger J.W., Carman K.R., Nisbet R.M. Indirect effects of contaminants in aquatic ecosystems. Science of the Total Environment, 2003; 317: 207–233 10.1016/S0048-9697(03)00141-4 14630423

[35] Kunugi M., Satoh S., Ihara K., Shibata K., Yamagishi Y., Kogame K., Obokata J., Takabayashi A., Tanaka A. Evolution of green plants accompanied changes in light-harvesting systems. Plant Cell Physiol. 2016; 57(6): 1231–1243. 10.1093/pcp/pcw071 27057002

[36] Wilde, FD (Ed.). Cleaning of equipment for water sampling (ver. 2.0), in: U.S. Geological Survey Techniques of Water-Resources Investigations, Book 9, 2004.

[37] Guillard RRL, Lorenzen CJ. Yellow-green algae with chlorophyllidae C12. J. Phycol. 1972; 8, 10–14.

[38] Nyholm N. and Kallqvist T. Methods for growth inhibition toxicity tests with freshwater algae. Environ. Toxicol. Chem. 1989; 8: 689–703.

[39] Franklin N.M., Stauber J.L., Apte S.C., Lim R.P. Effect of initial cell density on the bioavailability and toxicity of copper in microalgal bioassays. Environ. Toxicol. Chem. 2002; 21: 742–751. 11951947 10.1897/1551-5028(2002)021<0742:eoicdo>2.0.co;2

[40] Egorova EA, Drozdova IS, Bukhov NG. Modulating effect of far-red light on activities of alternative electron transport pathways related to Photosystem I. Russ. J. Plant Physiol. 2005; 52, 709–716.

[41] Hill R, Ralph PJ. Dark-induced reduction of the plastoquinone pool in zooxanthellae of scleractinian corals and implications for measurements of chlorophyll a fluorescence. Symbiosis (Rehovot) 2008; 46: 45–56.

[42] Ralph PJ, Hill R, Doblin MA, Davy SK. Theory and application of pulse amplitude modulated chlorophyll fluorometry in coral health assessment, in: Diseases of Coral. John Wiley & Sons, Inc, 2004; Hoboken, NJ, pp. 506–523.

[43] Figueroa F, Conde-Alvarez R, Gomez I. Relations between electrion transport rates determined by pulse amplitude modulated chlorophyll fluorescence and oxygen evolution in macroalgae under different light conditions. Photosynth. Res. 2003; 75, 259–275. 10.1023/A:1023936313544 16228606

[44] White EM, Kieber DJ, Sherrard J, Miller WL, Mopper K. Carbon dioxide and carbon monoxide photoproduction quantum yields in the Delaware Estuary. Mar. Chem. 2010; 118: 11–21.

[45] Weibull W. A statistical distribution function of wide applicability. ASME J. Appl. Mechanics, Trans. Am. Soc. Mech. Engin. 1951: 293–297.

[46] Platt T, Gallegos CL, Harrison WG. Photoinhibition of photosynthesis in natural assemblages of marine phytoplankton. J. Mar. Res. 1980; 38: 687–701.

[47] Azov Y. Effect of pH on inorganic carbon uptake in algal cultures. Appl. Environ. Microbiol. 1982; 43: 1300–1306. 16346029 10.1128/aem.43.6.1300-1306.1982 PMC244231

[48] Talmy D, Blackford J, Hardman-Mountford NJ, Polimene L, Follows MJ, Geider RJ. Flexible C:N ratio enhances metabolism of large phytoplankton when resource supply is intermittent. Biogeosciences Discuss. 2014; 11: 5179–5214.

[49] Bridges HR, Jones AJY, Pollak MN, Hirst J. Effects of metformin and other biguanides on oxidative phosphorylation in mitochondria. Biochem. J. 2014; 462: 475–487. 10.1042/BJ20140620 25017630 PMC4148174

[50] Wang D-S, Jonker JW, Kato Y, Kusuhara H, Schinkel AJ, Sugiyama Y. Involvement of organic cation transporter 1 in hepatic and intestinal distribution of metformin. J. Pharmacol. Exp. Ther. 2002; 302: 510–515. 10.1124/jpet.102.034140 12130709

[51] Bridges HR, Sirvio VA, Agip A-NA, Hirst J. Molecular features of biguanides required for targeting of mitochondrial respiratory complex I and activation of AMP-kinase. BMC Biology 2016; 14:65 10.1186/s12915-016-0287-9 27506389 PMC4977651

[52] He L, Wondisford FE. Metformin action: concentrations matter. Cell Metab. 2015; 21:159–62. 10.1016/j.cmet.2015.01.003 25651170

[53] Beardall J, Morris I. The concept of light intensity adaptation in marine phytoplankton: some experiments with *Phaeodactylum tricornutum*. Mar. Biol. 1976; 37: 377–387.

[54] Falkowski PG, Owens TG. Effects of light intensity on photosynthesis and dark respiration in six species of marine phytoplankton. Mar. Biol. 1978; 45: 289–295.

[55] Richardson K, Beardall J, Raven JA. Adaptation of unicellular algae to irradiance: An analysis of strategies. New Phytol. 1983; 93: 157–191.

[56] Suggett D.J., Moore C.M., Hickman A.E., Geider R.J. Interpretation of fast repetition rate (FRR) fluorescencce: signatures of phytoplankton community structure versus physiological state. Mar. Ecol. Prog. Ser. 2009; 376: 1–19.

[57] Neuwoehner J., Fenner K., Escher B.I. Physiological modes of action of fluoxetine and its human metabolites in algae. Environm. Sci. Tech. 2009; 43: 6830–6837. 10.1021/es9005493 19764256

[58] Muller P., Li X-P., Niyogi K.K. Non-photochemical quenching. A response to excess light energy. Plant Phys. 2001; 125: 1558–1566. 10.1104/pp.125.4.1558 PMC1539381 11299337

[59] Milligan A.J., Aparicio U.A., Behrenfeld M.J. Fluorescence and nonphotochemical quenching responses to simulated vertical mixing in the marine diatom *Thalassiosira weissflogii*. Mar. Ecol Prog Ser 2012; 448: 67–78.

[60] Peers G., Truong T.B., Ostendorf E., Busch A., Elrad D., Grossman A.R., et al An ancient light-harvesting protein is critical for the regulation of algal photosynthesis. Nature, 2009: 462: 518–522. 10.1038/nature08587 19940928

[61] Gilmore A.M., Hazlett T.L., Debrunner P.G., Govindjee. Comparative time-resolved photosystem II chlorophyll *a* fluorescence analyses reveal distinctive differences between photoinhibitory reaction center damage and xanthophyll cycle-dependent energy dissipation. Photochem. Photobiol. 1996; 64: 552–563. 8806231 10.1111/j.1751-1097.1996.tb03105.x

[62] Baena-González E, Rolland F, Thevelein JM, Sheen J. A central integrator of transcription networks in plant stress and energy signalling. Nature 2007; 448: 938–942. 10.1038/nature06069 17671505

[63] Zhang Y, Primavesi LF, Jhurreea D, Andralojc PJ, Mitchell RAC, Powers SJ, et al Inhibition of SNF1-Related Protein Kinase1 activity and regulation of metabolic pathways by trehalose-6-phosphate. Plant Physiol. 2009; 149: 1860–1871. 10.1104/pp.108.133934 19193861 PMC2663748

[64] Crozet P., Margalha L., Confraria A., Rodrigues A., Martinho C., Adamo M., et al Mechanisms of regulation of SNF1/AMPK/SnRK1 protein kinases. Front. Plant. Sci. 2014; 5: article 190, 1–17. 10.3389/fpls.2014.00190 PMC4033248 24904600

[65] Emanuelle S., Doblin M.S., Stapleton D.I., Bacic A., Gooley P.R. Molecular insights into the enigmatic metabolic regulator, SnRK1. Trends Plant Sci., 2016; 21: 341–353. 10.1016/j.tplants.2015.11.001 26642889

[66] Baena-Gonzalez E., Sheen J. Convergent energy and stress signaling. Trends Plant. Sci. 2008; 13: 474–482. 10.1016/j.tplants.2008.06.006 18701338 PMC3075853

[67] Lehti-Shiu MD, Shiu S-H. Diversity, classification and function of the plant protein kinase superfamily. Philos. Trans. R. Soc. B Biol. Sci. 2012; 367: 2619–2639. 10.1098/rstb.2012.0003 PMC3415837 22889912

[68] van Dam TJP, Zwartkruis FJT, Bos JL, Snel B. Evolution of the TOR pathway. J. Mol. Evol. 2011; 73: 209–220. 10.1007/s00239-011-9469-9 22057117 PMC3236823

[69] Serfontein J, Nisbet RER, Howe CJ, de Vries PJ. Evolution of the TSC1 / TSC2-TOR signaling pathway. Sci Signal. 2014; 3(128): 1–7. 10.1126/scisignal.2000803 20587805

[70] Polge C, Thomas M. SNF1/AMPK/SnRK1 kinases, global regulators at the heart of energy control? Trends Plant Sci. 2007; 12: 20–28. 5 10.1016/j.tplants.2006.11.005 17166759

[71] Hedbacker K. SNF1/AMPK pathways in yeast. Front. Biosci. 2008; 13: 2408 17981722 10.2741/2854 PMC2685184

[72] Wurzinger B., Nukarinen E., Nagele T., Weckwerth W., Teige M. The SnRK1 kinase as central mediator of energy signaling between different organelles. Plant. Phys. 2018; 176: 1085–1094. 10.1104/pp.17.01404 PMC5813556 29311271

[73] Cardol P., Gloire G., Havaux M., Remacle C., Matagne M., Franck F. Photosynthesis and state transitions in mitochondrial mutants of Chlamydomonas reinhardtii affected in respiration. Plant. Physiol. 2003; 133: 2010–2020. 10.1104/pp.103.028076 14630958 PMC300752

[74] Allen A.E., Laroche J., Maheswari U, Lommer M., Schauer N., Lopez P.J., et al Whole-cell response of the pennate diatom *Phaeodactylum tricornutum* to iron starvation. Proc. Natl. Acad. Sci. USA 2008; 105: 10438–10443. 10.1073/pnas.0711370105 18653757 PMC2492447

[75] Cardol P., Forti G., Finazzi G. Regulation of electron transport in microalgae. Biochim. Biophys. Acta 2011; 1807: 912–918. 10.1016/j.bbabio.2010.12.004 21167125

[76] Hirst J. Mitochondrial complex I. Ann. Rev. Biochem. 2013; 82: 551–575. 10.1146/annurev-biochem-070511-103700 23527692

[77] Dykens JA, Jamieson J, Marroquin L, Nadanaciva S, Billis PA, Will Y. Biguanide-induced mitochondrial dysfunction yields increased lactate production and cytotoxicity of aerobically-poised HepG2 cells and human hepatocytes in vitro. Toxicol. Appl. Pharm. 2008; 233: 203–210. 10.1016/j.taap.2008.08.013 18817800

[78] Brooks B.W., Riley T.M., Taylor R.D. Water quality of elluent-domninated ecosystems: ecotoxicological, hydrological, and management considerations. Hydrobiologia, 2006; 556: 365–379.

[79] Muir D., Simmons D., Wang X., Peart T., Villella M., Miller J., et al Bioaccumulation of pharmaceuticals and personal care product chemicals in fish exposed to wastewater effluent in an urban watershed. Sci. Rep. 2017; 7: 16999 10.1038/s41598-017-15462-x 29208974 PMC5717258

[80] Xie Z., Lu G., Liu J., Yan Z., Ma B., Zhang Z., et al Occurrence, bioaccumulation, and trophic magnification of pharmaceutically active compounds in Taihu Lake, China. Chemosphere, 2015; 138: 140–147. 10.1016/j.chemosphere.2015.05.086 26070079

[81] Xie Z., Lu G., Yan Z., Liu J., Want P., Wang Y. Bioaccumulation and trophic transfer of pharmaceuticals in food webs from a large freshwater lake. Environ. Poll. 2017; 222: 356–366. 10.1016/j.envpol.2016.12.026 28034558

[82] Baselga-Cervera B., Lopez-Rodas V., Balboa G., Huertas Cabilla E.I., Costas E. Mechanisms of rapid adaptation to environmental stressors in phytoplankton. J. Environ. Anal. Toxicol. 2016; 6: 5 no. 1000405.

[83] Bell G., Gonzalez A. Adaptation and evolutionary rescue in metapopulations experiencing environmental deterioration. Science 2011; 332: 1327–1330. 10.1126/science.1203105 21659606

[84] Lopez-Rodas V., Agrelo M., Carrillo E. Ferrero L., Larrauri A. et al Resistance of microalgae to modern water contaminants as the result of rare spontaneous mutations. Eur. J. Phycol. 2001; 36: 179–190.

[85] Carrera-Martinez D., Mateos-Sanz A., Lopez-Rodas V., Costas E. Adaptation of microalgae to a gradient of continuous petroleum contamination. Aquat. Toxicol 2011; 101: 342–350. 10.1016/j.aquatox.2010.11.009 21216344

[86] Miller T.H., Bury N.R., Owen S.F., MacRae J.I., Barron L.P. A review of the pharmaceutical exposome in aquatic fauna. Environ. Poll. 2018; 239: 129–16. 10.1016/j.envpol.2018.04.012 PMC5981000 29653304

